# Assessment of Image Quality of Coronary CT Angiography Using Deep Learning-Based CT Reconstruction: Phantom and Patient Studies

**DOI:** 10.3390/diagnostics13111862

**Published:** 2023-05-26

**Authors:** Pil-Hyun Jeon, Sang-Hyun Jeon, Donghee Ko, Giyong An, Hackjoon Shim, Chuluunbaatar Otgonbaatar, Kihong Son, Daehong Kim, Sung Min Ko, Myung-Ae Chung

**Affiliations:** 1 Department of Radiology, Wonju Severance Christian Hospital, Wonju 26426, Republic of Korea; iromeo138@naver.com (P.-H.J.); leadledled@naver.com (S.-H.J.); onllon@naver.com (D.K.); koreacurry@naver.com (G.A.); 2 Medical Imaging AI Research Center, Canon Medical System, Seoul 08826, Republic of Korea; hackjoon.shim@kr.medical.canon; 3 Department of Radiology, Seoul National University College of Medicine, Seoul 03080, Republic of Korea; chukarad@gmail.com; 4 Medical Information Research Section, Electronics and Telecommunications Research Institute, Daejeon 34129, Republic of Korea; kihong@etri.re.kr; 5 Department of Radiological Science, Eulji University, Seongnam 13135, Republic of Korea; goldcollar011@eulji.ac.kr; 6 Department of Bigdata Medical Convergence, Eulji University, Seongnam 13135, Republic of Korea

**Keywords:** deep learning reconstruction (DLR), coronary computed tomography angiography (CCTA), image quality

## Abstract

Background: In coronary computed tomography angiography (CCTA), the main issue of image quality is noise in obese patients, blooming artifacts due to calcium and stents, high-risk coronary plaques, and radiation exposure to patients. Objective: To compare the CCTA image quality of deep learning-based reconstruction (DLR) with that of filtered back projection (FBP) and iterative reconstruction (IR). Methods: This was a phantom study of 90 patients who underwent CCTA. CCTA images were acquired using FBP, IR, and DLR. In the phantom study, the aortic root and the left main coronary artery in the chest phantom were simulated using a needleless syringe. The patients were classified into three groups according to their body mass index. Noise, the signal-to-noise ratio (SNR), and the contrast-to-noise ratio (CNR) were measured for image quantification. A subjective analysis was also performed for FBP, IR, and DLR. Results: According to the phantom study, DLR reduced noise by 59.8% compared to FBP and increased SNR and CNR by 121.4% and 123.6%, respectively. In a patient study, DLR reduced noise compared to FBP and IR. Furthermore, DLR increased the SNR and CNR more than FBP and IR. In terms of subjective scores, DLR was higher than FBP and IR. Conclusion: In both phantom and patient studies, DLR effectively reduced image noise and improved SNR and CNR. Therefore, the DLR may be useful for CCTA examinations.

## 1. Introduction

According to the 2016 statistics of the American Heart Association, more than 15 million adults aged ≥20 years in the United States reported coronary heart disease [1]. In Korea, heart disease is also reported to be the second leading cause of death among chronic diseases after cancer [2]. Coronary computed tomography angiography (CCTA) is an important method that provides comprehensive morphological information about coronary artery stenosis, including the presence or absence of stenosis, the site of stenosis, the severity of stenosis, and characteristics of atherosclerotic plaques [3].

In CCTA, the main issues with image quality are noisy image quality in obese patients, blooming artifacts due to calcium and stents, high-risk coronary plaque, and radiation exposure to patients [4,5,6]. Various iterative image reconstruction algorithms have been developed to solve this problem [7,8]. According to the study results, iterative reconstruction (IR) reduces blooming artifacts and noise compared to filtered back projection (FBP). Compared to FBP, IR has significantly contributed to reducing the radiation dose and maintaining image quality.

Deep learning reconstruction (DLR) has recently been commercialized because of improvements in computer hardware and the development of artificial intelligence (AI). DLR is used for noise reduction in brain imaging [9], noise reduction in lung imaging [10], and abdominal low-dose CT [11]. In addition, DLR shows that the radiation dose can be reduced by approximately 40% in CCTA examinations [12]. FBP, which has been widely used as a standard, has the drawbacks of high noise and artifacts [13,14,15]. Hybrid iterative reconstruction (HIR) and model-based iterative reconstruction (MBIR) have been developed as alternatives to FBP [16,17,18]. HIR can reduce image noise and artifacts to some extent depending on the iterative blending ratio with FBP. However, a certain amount of image noise and artifacts remains in low-dose scan protocols [19]. The MBIR approach usually requires higher computational power and longer computation times. DLR has been successfully processed for image recognition, segmentation, and classification, and deep learning has demonstrated complex tasks on deep convolutional neural networks (DCNNs), which have shown remarkable performance in image classification as steps, enabling 1000 object types to be competently classified on more than 1 million images [20].

The Advanced Intelligent Clear-IQ Engine, from Canon Medical Systems, is the first commercialized deep learning reconstruction tool [21,22], and the DLR algorithm is taught to produce high-quality SNR images through an intense training process. The purpose of this study was to evaluate the effect of DLR on images in CCTA of CT with a 640-channel wide detector.

## 2. Materials and Methods

### 2.1. DLR

As a DLR tool, AiCE (Advanced Intelligent Clear-IQ Engine), a commercially available tool for CT, was used. In DLR, a DCNN compares the output image with a gold-standard reference image through the communication of neurons to preserve the spatial and low-noise characteristics of the MBIR algorithm. The DCNN uses a mathematically validated function to determine the amount of error between the output and reference datasets [23]. The more fluctuations there are in the data provided during training, the better the final algorithm performs in terms of quality and processing speed.

**Figure 1:**
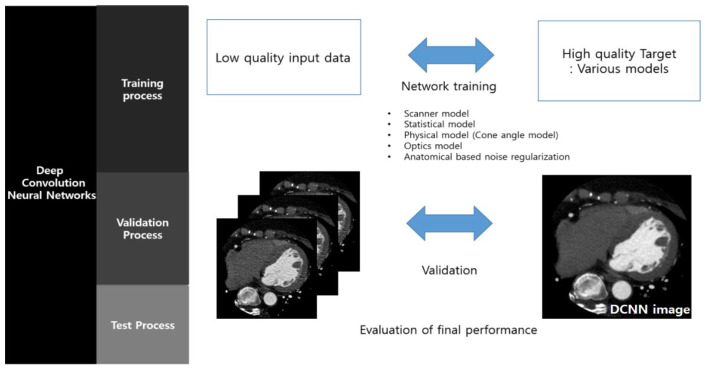
Process of deep learning reconstruction for DCNN.

As shown in Figure 1, AiCE is trained using thousands of pairs of data, one low quality and one high quality. The network learns to produce high-quality results from low-quality data compared to standard high-quality data [24].

### 2.2. Experimental Setup

Experiments were carried out in both the phantom and patient studies. A commercial CT scanner (Aquilion ONE GENESIS, Canon Medical Systems, Otawara-shi, Japan) capable of FBP, IR, and DLR was used for image acquisition. In this study, a tube voltage of 100 kV was used to reduce beam-hardening artifacts according to body mass index (BMI). Slice thickness is 0.5 mm, and image matrix is 512 × 512. After CT scan, images were reconstructed using FBP, IR, and DLR, respectively.

### 2.3. Phantom Study

**Figure 2:**
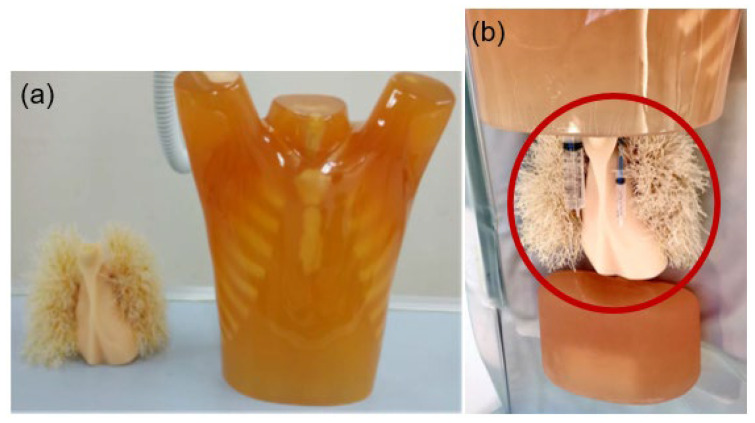
The lung phantom was used in this study. (**a**) Appearance of lung and heart model and chest model phantom. (**b**) Needle-free syringes to simulate the aortic root and the left main coronary artery are shown in the red circle.

Needle-free syringes (20 mL/cc and 5 mL/cc) were placed inside the chest phantom (Lungman, Kyoto Kagaku, Kyoto, Japan) to simulate the aortic root (AR) and left main coronary artery (LMCA) in the same manner as in the patient study (Figure 2b). Since the Hounsfield unit (HU) value was measured as the maximum value when only a pure contrast medium was used, the contrast medium and physiological saline were mixed in a 1:8 ratio and set at 600 HU (the average HU value of the AR and LMCA).

### 2.4. Patient Setup

We retrospectively reviewed the medical records of 90 patients who underwent CCTA between September 2020 and February 2021. This study was approved by the IRB committee of Wonju Severance Christian Hospital. The ages of the patients ranged from 38 to 86 years, with an average age of 61.8 years. The patients were divided into three groups according to BMI (kg/m^2^) (Table 1). The BMI distribution ranged from 18.40 kg/m^2^ to 31.18 kg/m^2^, with an average BMI of 24.54 kg/m^2^. For patients with a pre-examination heart rate (HR) of >65 bpm, 7.5 mg of Procoralan Tab Ivabradine (Betaloc; Les Laboratories Servier Industrie, Gidy, France) was administered orally, and 0.4 mg of nitroglycerin (Nitroquick, Ethex, MO, USA) was administered sublingually to all patients. Bolus tracking was used to scan the coronary artery when the image intensity (HU) value reached the target value by setting a region of interest (ROI) in the descending aorta at the bifurcation of the bronchi. CCTA scans were performed 2 cm above the carina to the diaphragm, excluding the entire aortic arch. Data were acquired in a cranio-caudal direction with a detector collimation of 0.5 × 80 mm, a gantry rotation time of 275 ms, a pitch of 0.813, a tube voltage of 100 kV for CCTA, and a tube current of 400–900 mA per rotation. A non-enhanced electrocardiography (ECG)-gated CCTA scan, prospectively triggered at 70–80% of the R–R interval, was performed to measure the coronary artery and aortic valve calcium score. ECG-based tube current modulation was used for CCTA, except in patients with a mean HR of >65 bpm or those with arrhythmias. A full-dose window of 20–70% of the cardiac cycle was used in patients with HR ≤80 bpm. For all CT examinations, a Stellant D dual-head power injector (Medrad) was used to administer a three-phase bolus at a rate of 4.5 mL/s. First, iopromide (70–80 mL) (Ultravist 370^®^, Bayer Healthcare, Berlin, Germany) was administered. Then, 45 mL of a 70–30% blend of a contrast medium and saline was administered. Finally, 45 mL of saline was administered. Detailed scan parameters are listed in Table 2. This study was classified into three groups according to BMI, and the images were reconstructed using three algorithms: FBP, IR, and DLR. All FBP, IR, and DLR CT images were excluded from beam-hardening artifact correction.

We also described the patients’ plaque distribution, stenosis severity, and calcium scores. Table 3 shows plaque distribution and stenosis severity, and Table 4 shows the calcium scores.

### 2.5. Image Analysis

Noise, CT density, the signal-to-noise ratio (SNR), and the contrast-to-noise ratio (CNR) were analyzed to evaluate images of AR and LMCA.

**Figure 3:**
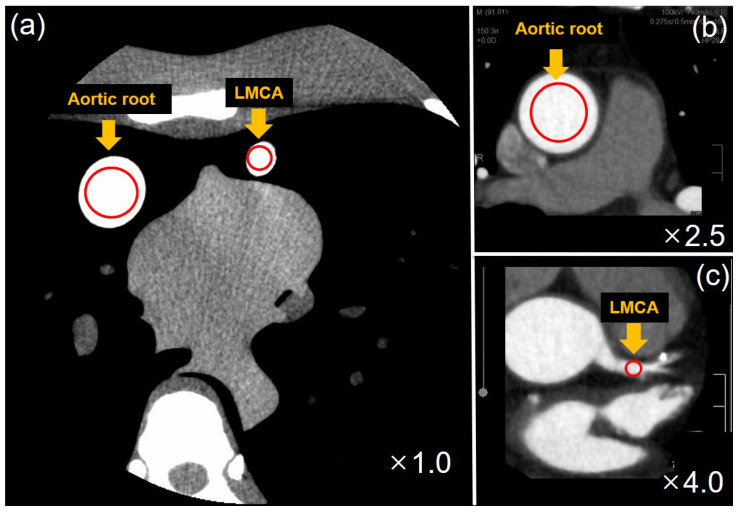
ROI is located in the aortic root (AR) and left main coronary artery (LMCA) in the lung phantom image (**a**), and ROI in the AR and LMCA in the patient image is shown in (**b**,**c**).

The image noise was measured as the standard deviation of the pixel value by measuring an ROI of 600 mm^2^ in the AR. CT density was measured at an ROI of 10 mm^2^ in a 5 mL/cc needleless syringe image. For the phantom image, the SNR was calculated using Equation (1).

(1) $$SNR=\frac{S}{SD}$$

where S is the average value of the pixels in the ROI of the simulated LMCA using a needleless syringe of 5 mL/cc. SD is a virtual AR made with a 20 mL/cc needleless syringe. The CNR was calculated using Equation (2).

(2) $$CNR=\frac{\left|S-B\right|}{SD}$$

where B is an ROI of 15 mm^2^ in the middle region of the heart of the phantom, which is close to the HU value of the actual epicardial fat. S and SD are the same values used for the SNR. The ROI in the phantom image for calculating noise, SNR, and CNR is shown in Figure 3a.

In the case of the patient image, the noise was measured as the standard deviation of an ROI of 600 mm^2^ at the AR. The CT density was measured as the average pixel value by setting an ROI of 10 mm^2^ in the LMCA. The SNR was measured using Equation (1). In the SNR equation, S is the average value of the ROI of the LMCA image, and SD is the standard deviation of the ROI of the AR.

The CNR was calculated using Equation (2). In the CNR equation, S is the average value of the ROI of the LMCA, B is the average value of the ROI of the epicardial fat, and SD is the standard deviation of the ROI of the AR. The ROI in the patient image for calculating noise, SNR, and CNR is illustrated in Figure 3b,c.

### 2.6. Dose Analysis

The effective dose was calculated using Equation (3).

(3) $$Effective\;Dose\left(mSv\right)=DLP\left(mGy\cdot cm\right)\times E_{DLP}(mSv\cdot {mGy}^{-1}\cdot {cm}^{-1})$$

The effective dose was calculated by multiplying the dose length product (DLP) by the effective dose ratio (E_DLP_) for chest examination per DLP recommended by EUR 16262 (0.017).

### 2.7. Subjective Image Analysis

Two experienced radiologists (with 5 and 21 years of experience in diagnostic radiology) independently evaluated image quality. The radiologists were blinded to both the image reconstruction and patient characteristics and randomly evaluated the CCTA images, and the results were averaged for a subjective analysis. A five-point Likert scale was used for the image analysis for the following three aspects: overall image quality, image noise, and proximal vessels (Table 5).

### 2.8. Statistical Analysis

All data were analyzed using independent sample tests (SPSS software, version 25.0, Armonk, NY, USA), and the Kruskal–Wallis H test (three groups) and Wilcoxon signed rank test (two groups) were used to test for normality. Statistical significance was set at *p* < 0.05.

## 3. Results

### 3.1. Phantom Study

**Figure 4:**
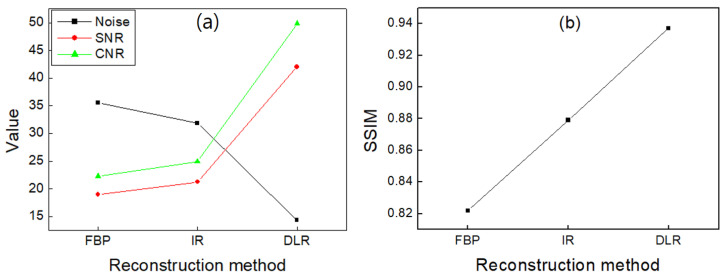
(**a**) Noise, SNR, and CNR values, and (**b**) SSIM in phantom images reconstructed by FBP, IR, and DLR, respectively.

Figure 4a shows the results of the noise, SNR, and CNR values in the phantom images reconstructed by FBP, IR, and DLR. Compared with FBP and IR, the noise of DLR was reduced by 59.8% and 55.1%, respectively, and SNR was increased by 121.4% and 97.7%, respectively. The CNR of DLR increased by 123.6% compared to FBP, and the CNR of DLR increased by 100.2% compared to IR. As shown in Figure 4b, the structural similarity index measure (SSIM) was measured for FBP, IR, and DLR. The reference image was used by averaging the pixel values of 20 FBP images of the phantom. The SSIM of water was 0.82, 0.88, and 0.94 for FBP, IR, and DLR, respectively.

### 3.2. Patient Study

In the phantom results, DLR showed the best quality in terms of noise, SNR, and CNR, and based on the results, patient images were acquired because quantification of patient image quality was also necessary in clinical practice. In the patient study, there were no significant differences in the CT density of the LMCA images in FBP, IR, and DLR (*p* = 0.713). The noise was measured in the AR, and SNR was measured in the LMCA and AR. CNR was measured in the LMCA and epicardial fat. According to the noise results, in Group A (BMI < 22), DLR reduced noise by 45.8% and 33.1%, respectively, compared to FBP and IR. In Group B (22 < BMI < 26), DLR reduced noise by 51.8% and 34.5%, respectively, compared to FBP and IR. Moreover, in Group C (26 < BMI), DLR reduced noise by 54.6% and 35.8%, respectively, compared to FBP and IR (*p* < 0.001).

The SNR results confirmed that the DLR was higher than that of the FBP and IR methods. In Group A, DLR increased SNR by 76.4% and 44.1%, respectively, compared with FBP and IR. In Group B, DLR increased SNR by 92.9% and 38.8%, respectively, compared with FBP and IR. In Group C, DLR increased SNR by 104.9% and 49.3%, respectively, compared with FBP and IR (*p* < 0.001).

According to the CNR results, DLR increased CNR compared to FBP and IR. In Group A, DLR increased CNR by 22.4% and 39.6%, respectively, compared with FBP and IR. In Group B, DLR increased CNR by 31.5% and 43.9%, respectively, compared with FBP and IR. In Group C, DLR increased CNR by 37.2% and 45.5%, respectively, compared with FBP and IR (*p* < 0.001).

**Figure 5:**
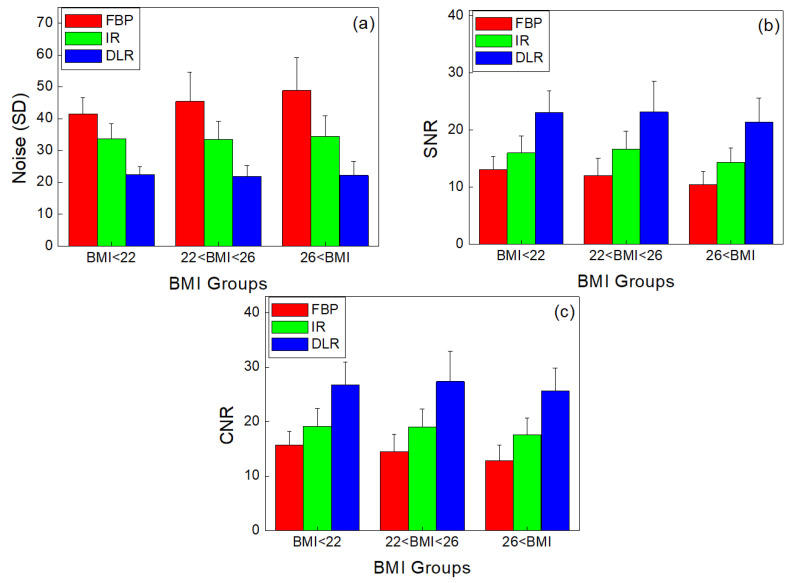
(**a**) Noise, (**b**) SNR, and (**c**) CNR values according to BMI group in patient images reconstructed by FBP, IR, and DLR, respectively (*p* < 0.001).

Figure 5 shows the noise, SNR, and CNR of FBP, IR, and DLR for the CT images of the three groups of patients.

In Group A (BMI < 22), the mean CTDIvol was 27.1 mGy, and the average DLP was 416.7 mGy·cm. In Group B (22 < BMI < 26), the mean CTDIvol was 23.3 mGy, and the mean DLP was 346.9 mGy·cm. In Group C (26 < BMI), the mean CTDIvol was 27.4 mGy, and the mean DLP was 397.0 mGy·cm. The average effective dose was 7.1 mSv, 5.9 mSv, and 6.8 mSv in Groups A, B, and C, respectively (Table 6).

**Figure 6:**
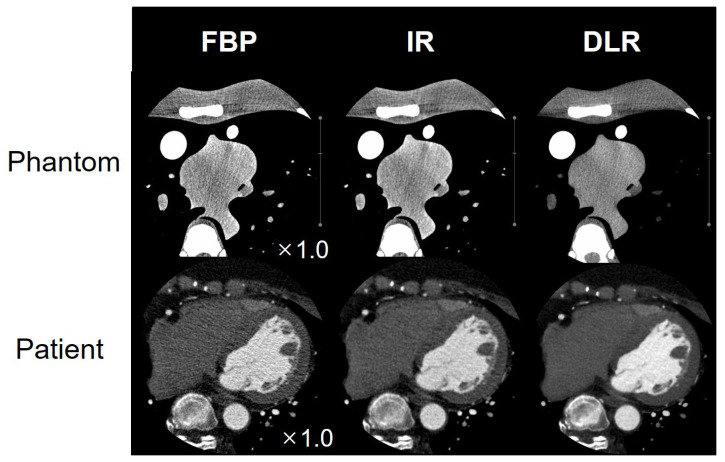
Phantom and patient images of CCTA reconstructed by FBP, IR, and DLR, respectively.

**Figure 7:**
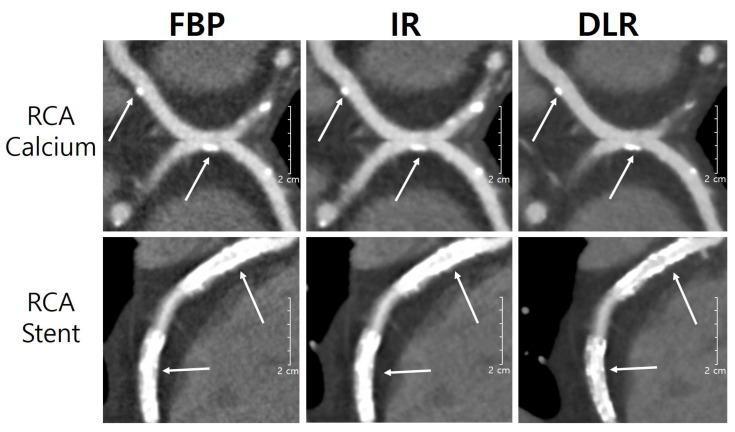
Right coronary artery image with calcium (upper row) and stent (bottom row) reconstructed using FBP, IR, and DLR. Compared to FBP and IR images, the DLR image has low noise and reduces blooming artifacts.

Examples of phantom and patient FBP, IR, and DLR axial images are shown in Figure 6. The DLR’s image quality has been improved, such as noise reduction in both phantom and patient images. Figure 7 shows a right coronary artery (RCA) image with calcium and a stent reconstructed using FBP, IR, and DLR. DLR shows better image quality than FBP and IR. In the patient image results, DLR reduced noise compared to FBP and IR. In addition, DLR reduced blooming artifacts in the calcium and stent images.

### 3.3. Subjective Image Analysis

The overall image quality, image noise, and proximal vessels of both observers are summarized in Table 7. The average overall image quality score was higher for DLR (4.8 ± 0.4) than for FBP (3.6 ± 0.6) and IR (4.3 ± 0.5) (*p* < 0.001). Image noise was scored higher in DLR (4.8 ± 0.4) compared with FBP (3.5 ± 0.6) and IR (4.3 ± 0.6) (*p* < 0.001). The scores for the proximal vessels were greater for DLR (4.9 ± 0.4) than for FBP (3.8 ± 0.5) and IR (4.3 ± 0.5) (*p* < 0.001).

## 4. Discussion

Our study showed that DLR produced improved image quality for CCTA images compared to FBP and IR in both phantom and patient quantitative evaluations.

In the phantom study, DLR significantly improved the image quality compared with FBP and IR. SNR and CNR are widely used to evaluate the contrast enhancement of blood vessels, and SNR, CNR, and noise were also evaluated in this study [25]. Regarding quantitative imaging metrics, DLR increased SNR and CNR compared with FBP and IR. Compared with FBP and IR, the SNR of DLR increased by 121.4% and 97.7%, respectively. The CNR of DLR increased by 123.6% compared to FBP, and the CNR of DLR increased by 100.2% compared to IR. In addition, DLR reduced image noise compared to FBP and IR in terms of image quality. As a result, in the phantom study, DLR reduced noise by 59.8% and 55.1% compared with FBP and IR, respectively.

In patient studies, DLR also showed an improvement in image quality compared to FBP and IR. Regardless of the BMI category in patient results, DLR improved SNR and CNR compared to FBP and IR (*p* < 0.001). In addition, DLR also reduced noise compared to FBP and IR (*p* < 0.001), regardless of the BMI category. Similarly, the DLR tended to have higher subjective image quality scores. In addition, DLR decreased image noise compared to FBP and IR and achieved high blood vessel attenuation and clear vessel wall definition. The radiation dose in Group C (obese) was significantly higher than Group A (about two-fold), and age and lifetime attributable risk (LAR) should be considered when choosing an imaging modality in clinical examinations for individual patients.

The main limitation of FBP is that it is very sensitive to noise, as it does not account for the Poisson distribution or photon number statistics. Therefore, FBP needs to increase the radiation dose to lower the noise in the CT image. Although IR reduces high-frequency noise components, its performance is limited in terms of reducing low-frequency noise components, resulting in an unnatural image appearance, especially at low doses. The DLR effectively suppresses low-spatial-frequency noise, producing a desirable noise texture. Therefore, DLR shows better image characteristics than FBP and IR in terms of noise magnitude, noise texture, spatial resolution, and low-contrast discrimination [26,27]. Figure 7 shows the image quality for image reconstruction of the phantom and patient images. Although this study did not evaluate diseases such as coronary artery stenosis, DLR achieved qualitative improvements in image quality and significant noise reduction. Our work confirms that deep learning-based denoising techniques can further improve image quality in the routine clinical setting of CCTA.

DLR has potential applications in various clinical settings, such as cardiac CT scans using ECG-gated tube current modulation. Considering the noise reduction, SNR, and CNR improvements in CCTA imaging, DLR would allow low-dose acquisition protocols. Additional research is needed to consider the possibility of reducing the radiation dose and how to use the reduced dose for tube current modulation.

CCTA has been applied noninvasively to monitor plaque progression, evaluation of plaque morphology, and quantification of plaques to evaluate atherosclerosis due to radiation dose reduction [28]. Due to these advantages, the diagnostic accuracy of CCTA has been evaluated in several studies, and the potential application of CCTA to coronary artery disease in clinical practice has been reported. In particular, blooming artifacts in CT images due to severe calcium levels affect plaque quantification. Figure 7 shows the possibility of reducing blooming artifacts by maintaining smooth margins while providing superior spatial resolution for calcium and stent imaging. Evaluation of the coronary artery stent image can be achieved by analyzing the profile of the stent present in the blood vessel [29]. In this study, CCTA’s DLR image was evaluated to be both quantitatively and qualitatively superior to FBP and IR in image restoration. In future studies, we intend to include those that contribute to the reduction of blooming artifacts in DLR and plaque quantification.

Our study confirmed the potential of DLR to improve image quality and reduce the radiation dose in the coronary arteries arising from the cardiovascular system. The use of CCTA with reconstruction methods, such as FBP and IR, may have limitations depending on the patient. In particular, calcification of blood vessels may increase the frequency of invasive coronary angiography due to the overestimation of lesions [30]. DLR-based CCTA examination can show excellent contrast and plaque characterization due to noise reduction, increased image homogeneity, and artifact reduction effects. Through this, an accurate evaluation of stenosis severity will be possible, and CCTA will become a noninvasive imaging tool. Based on this, it will be able to play an important role in the future in enabling the automatic analysis of vascular diseases through AI.

## 5. Conclusions

This study showed that DLR is very effective in reducing image noise and improving the parameters of images, such as SNR and CNR, compared to conventional FBP and IR images. DLR has the potential to maintain image quality at low doses.

## Figures and Tables

**Table 1:** Three patient groups according to body mass index.

Patient Characteristics	Group A * BMI < 22	Group B 22 ≤ BMI ≤ 26	Group C BMI > 26
No. of Patients	24	31	35
No. of Females/Males	10/14	18/13	24/11
Age (Years)	54.80	64.41	63.53
BMI (kg/m^2^)	20.11	24.42	28.33

* BMI: Body mass index.

**Table 2:** Imaging conditions in this study.

Scan Parameters	Scan Conditions
Tube voltage (kV)	100
Tube current (mA)	400–900
Slice thickness (mm)	0.5
Pitch	0.813
Time resolution (s)	0.135
R–R interval (%)	70–80
Image matrix	512 × 512
Reconstruction method	^1^ FBP, ^2^ IR, ^3^ DLR

^1^ FBP: Filtered back projection. ^2^ IR: Iterative reconstruction. ^3^ DLR: Deep learning reconstruction.

**Table 3:** The characteristics of coronary artery for plaque distribution and stenosis severity.

Plaque Distribution/ Stenosis Severity	1~25%	26~50%	21~75%	76~100%	*p* Value
RCA	71	19	7	3	0.001
LM	75	21	5	2	0.001
LCA	70	18	4	1	0.001
LCX	76	11	3	0	0.001

**Table 4:** The degree of coronary artery by absolute calcium score in this study.

Calcium Score	0 (Absent)	1~100 (Discrete)	100~400 (Moderate)	401 or Higher (Accentuated)	*p* Value
RCA	48	29	10	3	0.001
LM	43	30	15	2	0.001
LCA	52	28	16	4	0.001
LCX	60	21	8	1	0.001

**Table 5:** Scoring for subjective image analysis.

Score	Overall Image Quality	Image Noise	Proximal Vessel
5	Excellent	Minimal	High vessel attenuation and clear vessel wall
4	Good	Average	Good vessel attenuation and well preserved vessel wall
3	Moderate	Moderate	Adequate vessel attenuation and moderate vessel wall
2	Poor	Marked	Low vessel attenuation and blurring of vessel wall
1	Bad	Severe	Inadequate vessel attenuation and poor vessel wall

**Table 6:** Radiation doses of the three patient groups in the patient study.

Dose Parameters	Group A BMI < 22	Group B 22 < BMI < 26	Group C 26 < BMI	*p* Value
CTDI_vol_ (mGy)	16.38	18.2	34.8	0.001
DLP (mGy*cm)	273.4	338.1	446.8	0.001
Effective dose (mSv)	4.6	5.8	7.6	0.001

**Table 7:** Subjective image analysis for image reconstruction methods.

	Overall Image Quality	Image Noise	Proximal Vessels
FBP	3.6 ± 0.6	3.5 ± 0.6	3.8 ± 0.5
IR	4.3 ± 0.5	4.3 ± 0.6	4.3 ± 0.5
DLR	4.8 ± 0.4	4.8 ± 0.4	4.9 ± 0.4
*p* value	0.001	0.001	0.001

## Data Availability

Not applicable.
